# Draft genome sequence of a *Fusobacterium nucleatum* strain isolated from a patient in Kazakhstan with colorectal cancer

**DOI:** 10.1128/MRA.00367-23

**Published:** 2023-09-15

**Authors:** Gulmira Kulmambetova, Talgat Utupov, Botakoz Kurentay, Dana Auganova, Pavel Tarlykov, Alexandr Shevtsov, Asset Daniyarov, Saule Khamzina, Meiram Mamlin, Arman Kozhakhmetov, Sanzhar Shalekenov

**Affiliations:** 1 Genomic Laboratory, National Center for Biotechnology, Astana, Kazakhstan; 2 National Laboratory Astana, Nazarbayev University, Astana, Kazakhstan; 3 Multidisciplinary Surgery Center, National Research Oncology Center, Astana, Kazakhstan; 4 Department of Surgery, Nazarbayev University School of Medicine, Astana, Kazakhstan; Wellesley College Department of Biological Sciences, Wellesley, Massachusetts, USA

**Keywords:** *Fusobacterium nucleatum*, genome sequence, colorectal cancer

## Abstract

*Fusobacterium nucleatum* is an invasive obligate anaerobe found in the oral microbiota and associated with colorectal cancer. Here, we announce the draft genome sequence of *Fusobacterium nucleatum* strain Fn11kaz from a patient with colorectal cancer in Kazakhstan.

## ANNOUNCEMENT

*Fusobacterium nucleatum* is a Gram-negative anaerobic bacillus that is a significant contributor to colorectal cancer (CRC). This opportunistic pathogen may colonize colon tissues via the bloodstream. Recent data have shown that the tumor microenvironment is enriched with *F. nucleatum* (1, 2). *F. nucleatum* isolated from colon cancer tissue has shown the presence of virulence factors, such as adhesins and hemagglutinin (a galactose-binding lectin) (3, 4). However, the etiology of CRC is complex, and various factors play a major role in its pathogenesis.

A biopsy sample was collected from the colon cancer tissue of a patient with colorectal cancer in November 2022 at the National Research Oncology Center, Astana, Kazakhstan. Informed consent and questionnaires were approved by the Local Ethics Committee of the RSE National Center for Biotechnology of the Ministry of Healthcare of the Republic of Kazakhstan (extract from protocol no. 1 of 1 April 2022). The biopsy sample, placed on a 20% sucrose solution, was transported to the laboratory within 2 hours. Once in the laboratory, the biopsy material was homogenized, and 80 µL of the homogenized solution was cultivated on Columbia agar (5% defibrinated sheep blood, vitamin K, hemin, josamycin, vancomycin, and neomycin) and incubated for 72 hours under anaerobic conditions in an AnaeroGen system (reference no. AN0035A) at 37°C. The taxonomic classification of the *F. nucleatum* strain Fn11kaz was identified using 16S rRNA gene sequence analysis and the MALDI Biotyper microbial identification system (Bruker). The 16S rRNA sequences showed a 99.33% similarity of the V1-V4 gene region fragment to the *F. nucleatum* reference strain (NCBI accession number CP117525.1). The strain was stored at −70°C in a cryoprotective medium. DNA was extracted using the cetyltrimethylammonium bromide procedure (5). An Illumina MiSeq platform was used to carry out whole-genome sequencing, and a MiSeq reagent v3 600-cycles cartridge was used. DNA libraries were prepared using an Illumina DNA Prep Kit. The genome of *F. nucleatum* strain Fn11kaz has a length of 2,134,385 bp. It was sequenced with 61-fold coverage, a mean read length of 271 bp, and a G + C content of 26.91%. A total of 762,209 reads were produced. The quality check of the reads was performed using FastQC v.0.11.9 (6). A total of 34 contigs, with an N_50_ value of 199,090 bp, were *de novo* assembled using SPAdes v.3.1.0 (7). The final assemblies were annotated with the NCBI Prokaryotic Genome Annotation Pipeline (PGAP) v.6.5 (8). Default parameters were used for all software. Annotation of the Fn11kaz genome with PGAP identified a total of 2,031 genes, of which 1,963 were predicted coding sequences, including the vanTG gene, which causes resistance to glycopeptide antibiotics. Additionally, there were 43 tRNAs, 3 ncRNAs, and 1 complete 23S rRNA gene.

## Data Availability

This Whole Genome Shotgun project has been deposited at DDBJ/ENA/GenBank under the accession JARWBA000000000. The version described in this paper is version JARWBA010000000. The raw data from BioProject no. PRJNA954179 were submitted to the NCBI Sequence Read Archive under experiment accession no. SRR24390575.
